# Sex-based differential regulation of oxidative stress in the vasculature by nitric oxide

**DOI:** 10.1016/j.redox.2015.01.007

**Published:** 2015-01-13

**Authors:** Rommel C. Morales, Edward S.M. Bahnson, George E. Havelka, Nadiezhda Cantu-Medellin, Eric E. Kelley, Melina R. Kibbe

**Affiliations:** a Division of Vascular Surgery, Northwestern University, Chicago, IL, USA; b Simpson Querrey Institute for BioNanotechnology, Northwestern University, Chicago, IL, USA; c Vascular Medicine Institute, University of Pittsburgh, Pittsburgh, PA, USA; d Jesse Brown Veterans Affairs Medical Center, Chicago, IL, USA

**Keywords:** Neointimal hyperplasia, Superoxide, Nitric oxide, Vascular, Sex differences

## Abstract

**Background:**

Nitric oxide (^•^NO) is more effective at inhibiting neointimal hyperplasia following arterial injury in male versus female rodents, though the etiology is unclear. Given that superoxide (O_2_^•−^) regulates cellular proliferation, and ^•^NO regulates superoxide dismutase-1 (SOD-1) in the vasculature, we hypothesized that ^•^NO differentially regulates SOD-1 based on sex.

**Materials and methods:**

Male and female vascular smooth muscle cells (VSMC) were harvested from the aortae of Sprague-Dawley rats. O_2_^•−^ levels were quantified by electron paramagnetic resonance (EPR) and HPLC. *sod-1* gene expression was assayed by qPCR. SOD-1, SOD-2, and catalase protein levels were detected by Western blot. SOD-1 activity was measured via colorimetric assay. The rat carotid artery injury model was performed on Sprague-Dawley rats ±^•^NO treatment and SOD-1 protein levels were examined by Western blot.

**Results:**

*In vitro*, male VSMC have higher O_2_^•−^ levels and lower SOD − 1 activity at baseline compared to female VSMC (*P* < 0.05). ^•^NO decreased O_2_^•−^ levels and increased SOD − 1 activity in male (*P*<0.05) but not female VSMC. ^•^NO also increased *sod*− *1* gene expression and SOD − 1 protein levels in male (*P*<0.05) but not female VSMC. *In vivo*, SOD-1 levels were 3.7-fold higher in female versus male carotid arteries at baseline. After injury, SOD-1 levels decreased in both sexes, but ^•^NO increased SOD-1 levels 3-fold above controls in males, but returned to baseline in females.

**Conclusions:**

Our results provide evidence that regulation of the redox environment at baseline and following exposure to ^•^NO is sex-dependent in the vasculature. These data suggest that sex-based differential redox regulation may be one mechanism by which ^•^NO is more effective at inhibiting neointimal hyperplasia in male versus female rodents.

## Highlights

• The baseline redox environment in the vascular is sex-dependent.
• Nitric oxide differentially affects the vascular redox environment between the sexes.
• Nitric oxide decreases superoxide (O_2_^.^) levels, by increasing SOD-1 activity, *sod1* gene expression and SOD-1 protein levels in male vascular smooth muscle cells, but not in females.
• Sex-based differential redox regulation may be one mechanism by which is more effective at inhibiting neointimal hyperplasia in male versus female rodents.

## Graphical abstract

**Figure:**
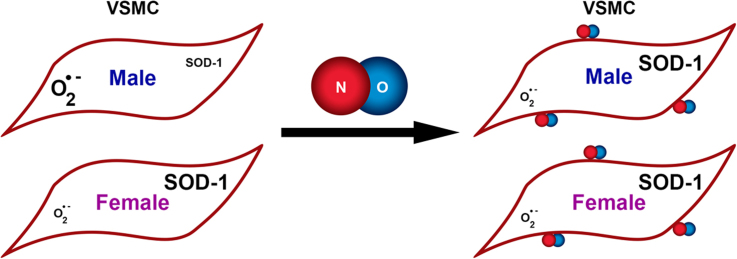

## Introduction

Neointimal hyperplasia limits the long-term durability of vascular interventions such as balloon angioplasty, stenting, endarterectomy, and bypass grafting. Current FDA approved drug eluting stents, designed to prevent the development of neointimal hyperplasia, deliver derivatives of two different classes of drugs (i.e., rapamycin and paclitaxel), both of which indiscriminately inhibit all cellular proliferation, including endothelial cell proliferation. Thus, there is a great need to develop novel therapies that effectively prevent neointimal hyperplasia while also promoting vascular healing. Nitric oxide (^•^NO) is one such drug that possesses many different vasoprotective properties. ^•^NO is a small gaseous molecule that is known to inhibit platelet adherence and aggregation, mitigate leukocyte chemotaxis, and prevent vascular smooth muscle cell (VSMC) and adventitial fibroblast proliferation and migration [1], [2], [3], [4], [5], [6], [7], [8], [9]. Simultaneously, ^•^NO stimulates endothelial cell proliferation and prevents endothelial cell apoptosis [10], [11]. Our laboratory and others, have demonstrated the beneficial effect of ^•^NO delivery to the vasculature to prevent neointimal hyperplasia in various different small and large animal models of arterial injury and bypass grafting [12], [13], [14], [15], [16], [17], [18], [19], [20]. However, our laboratory also demonstrated that ^●^NO has differential efficacy at inhibiting neointimal hyperplasia based on sex and hormone status [21]. The etiology for this difference, with ^●^NO being much more effective at inhibiting neointimal hyperplasia in males compared to females, and in hormonally intact versus castrated animals, remains unclear.

One possible explanation may reside in the regulation of oxidative stress between the sexes. Indeed, Vassalle et al. demonstrated differing levels of oxidative stress between men and postmenopausal women, with and without coronary artery disease (CAD). While postmenopausal females with CAD exhibited greater oxidative stress compared to men, they presented with less severe CAD [22]. Reactive oxygen species (ROS) have been found to extensively contribute to the severity of vascular disease and subsequent formation of neointimal hyperplasia [23], [24], [25]. Specifically, superoxide (O_2_^•−^), one of the main ROS, has been shown to be elevated after vascular injury, resulting in the formation of neointimal hyperplasia through increased proliferation and migration of VSMC and adventitial fibroblasts [26], [27], [28]. Superoxide dismutases, modulate this response through dismutation of O_2_^•−^ into oxygen and hydrogen peroxide, with the latter being enzymatically converted to water by catalase or other antioxidant peroxidases [29], [30]. ^•^NO can readily react with O_2_^•−^ to form peroxynitrite, which can have detrimental effects in the vasculature. Our laboratory recently demonstrated that ^•^NO regulates O_2_^•−^ levels in a cell-specific manner in the vasculature through modulating SOD-1 levels [31]. Thus, given the role of O_2_^•−^ in stimulating VSMC proliferation and migration and the role of ^•^NO in modulating neointimal hyperplasia and SOD-1, we hypothesize that ^•^NO differentially regulates SOD-1 levels based on sex. Here, we investigate the effect of ^•^NO on O_2_^•−^ generation, *sod-1 gene* expression, SOD-1 protein levels, and SOD activity *in vitro* and *in vivo* in male and female rodent models.

## Materials and methods

### ^•^NO-releasing donor

1-[N-(2-aminoethyl)-N-(2-ammonioethyl)amino]diazen-1-ium-1,2-diolate (DETA/NO) and disodium 1-[(2-carboxylato)pyrrolidine-1-yl]diazen-1-ium-1,2-diolate (PROLI/NO) were supplied by Dr. Larry Keefer (National Cancer Institute). Both DETA/NO and PROLI/NO are diazeniumdiolate ^•^NO donors that release 2 mols of ^•^NO per mole of compound at a predictable rate under physiologic conditions of pH 7 and temperature 37 °C [32]. Given that the duration of the *in vitro* experiments were up to 24 h, we used DETA/NO for these experiments since it has a half-life of 20 h. PROLI/NO was used for all animal studies given our prior work demonstrating superior efficacy of PROLI/NO in this animal model compared to other diazeniudiolate ^•^NO donors [17], [19], [21], [31].

### Rat carotid artery injury model

All animal procedures were performed in accordance with principles outlined in the Guide for the Care and Use of Laboratory Animals published by the National Institutes of Health (NIH Publication 85-23, 1996) and approved by the Northwestern University Animal Care and Use Committee. Adult male and female Sprague-Dawley rats (Harlan, Indianapolis, IN) weighing between 250–400 g were used for the study. Treatment groups included control, injury, and injury +^•^NO (*n*=5 rats/treatment group). Rats were anesthetized with inhaled isoflurane (0.5–2%), with subcutaneous atropine administration (0.1 mg/kg) to minimize airway secretions. The right common carotid artery for each rat served as the control group. Following sterile preparation, a midline neck incision was made. The left common, internal, and external carotid arteries were dissected, followed by occlusion of the internal and common carotid arteries. A No. 2 French arterial embolectomy catheter (Edwards Lifesciences, Irvine, CA) was inserted into the external carotid artery and advanced into the common carotid artery. The balloon was inflated to a pressure of 5 atm for 5 min to cause uniform injury. After the balloon was deflated and removed, the external carotid artery was ligated and blood flow restored. For the injury +^•^NO group, 10 mg of PROLI/NO was applied evenly to the external surface of the common carotid artery after balloon injury, as previously described [17], [19], [21], [31]. Following injury and treatment, neck incisions were closed. Rats were sacrificed 3 days after treatment to harvest carotid arteries. Arteries within treatment groups were pooled, frozen in liquid nitrogen, powdered with mortar and pestle, and homogenized in 20 mM Tris (pH 7.4) with 1 µM phenylmethylsulfonyl fluoride, 1 µM leupeptin, and 1 µM sodium orthovanadate (Sigma, St. Louis, MO). Western blot analysis was performed as described below.

### Cell culture

VSMC were harvested from the aortae of male and female Sprague-Dawley rats (Harlan) via methods as described by Gunther et al. [33] Male and female VSMC were confirmed via PCR (Supplementary Fig. 1) using SRY and GAPDH primers (IDT, Coralville, IA). Cells were maintained in media containing equal volumes of Ham’s F12 and Dulbecco’s modified Eagle’s medium – low glucose (DMEM) (Invitrogen, Carlsbad, CA), complemented with 100 units/mL penicillin (Corning, Corning, NY), 100 µg/mL streptomycin (Corning), 4 mM l-glutamine (Corning), and 10% fetal bovine serum (FBS) (Invitrogen). Cells were incubated at 37 °C with 5% CO_2_. To synchronize cells prior to DETA/NO treatment, cells were exposed to media lacking FBS for 24 h at 37 °C with 5% CO_2_. VSMC used in this study were between passage 3 and 9.

### EPR analysis

VSMC were plated on 100-mm dishes and allowed to attach overnight. Cells were serum starved for 24 h and exposed to DETA/NO (0.5–1.0 mM), pegylated (PEG)-SOD (50 U), or control media for 24 h. PEG-SOD was used as a control to ensure the signal measured was O_2_^•−^-dependent. Cells were then washed with cold PBS, incubated with the cell-permeable spin probe, 1-hydroxy-3-methoxy-carbonyl-2,2,5,5-tetramethlpyrrolidine (CMH) (Enzo Life Sciences, Ann Arbor, MI) for 30 min, and subsequently analyzed in a temperature- and O_2_-controlled Bruker EPR (Millerica, MA) at 37 °C, as described by Dikalov et al. [34]. The intensity of the first peak of the ^•^CM radical spectrum was quantified. EPR signal intensity was normalized per mg of protein. All buffers contained 25 µM deferoxamine and were treated with Chelex resin from Biorad (Hercules, CA) to minimize deleterious effects of possible contaminating metals.

### 2-Hydroxyethidium analysis

VSMC were plated on 100-mm dishes and were incubated overnight to facilitate attachment. After cells reached 80% confluence, VSMC were exposed to DETA/NO (0.5–1.0 mM), pegylated PEG-SOD (50 U), or control media for 24 h. PEG-SOD was used as a control to ensure the changes measured were O_2_^•−^-dependent. After 24 h of exposure to the various experimental conditions, VSMC were exposed to 10 µM of dihydroethidium (DHE) (Invitrogen) in the dark for 30 min. Subsequently, cells were washed with cold PBS, scraped, collected, protected from light, and stored at − 80 °C. O_2_^•−^ levels were determined by quantifying the levels of 2-hydroxyethidium (2-OH-E) by HPLC with electrochemical detection according to Zielonka et al. [35]. The results were expressed as pmols of O_2_^•−^ per mg of protein in total cell lysate.

### Western blot analysis

After 24 h of exposure to the various experimental conditions, VSMC were washed with cold PBS, scraped, and resuspended in 20 mM Tris at pH 7.4, supplemented with 1 µM phenylmethylsulfonyl fluoride, 1 µM leupeptin, and 1 µM sodium orthovanadate (Sigma). Protein concentrations were determined via a bicinchoninic acid protein assay per manufacturer’s instructions (Pierce, Rockford, IL). Equivalent protein amounts of each cell lysate were subjected to sodium dodecyl sulfate-polyacrylamide gel electrophoresis on 13% separating gels and transferred to nitrocellulose membranes (Schleicher & Shuell, Keene, NH). Membranes were probed with antibodies raised against either β-actin, SOD-1, SOD-2, or catalase for 1 h at room temperature or overnight at 4 °C, followed by hybridization with goat anti-rabbit secondary antibody conjugated to HRP for 1 h at room temperature. Proteins were imaged with chemiluminescent reagents, Supersignal Substrate, and processed according to manufacturer’s instructions (Pierce). Films exposed to membranes were analyzed with ImageJ v.138x (NIH, Bethesda, MD). Protein levels were determined by fold difference in density, standardized to male control, following normalization to β-actin loading controls.

### Quantitative PCR

After either 6 or 12 h of exposure to the various experimental conditions, VSMC were washed with cold PBS, scraped, and collected. RNA was isolated from samples via RNAeasy Protect Mini Kit per manufacturer’s instructions (Qiagen, Germantown, MD). RNA concentrations were determined via a Gen5 Take 3 Module per manufacturer’s instructions (BioTek, Winooski, VT). Equivalent RNA amounts were converted into cDNA via a QuantiTect Reverse Transcription Kit per manufacturer’s instructions (Qiagen). Quantitative PCR was performed via a QuantiTect SYBR Green PCR Kit using SOD-1 and GAPDH primers according to manufacturer’s instructions (Qiagen). Fluorescence was detected via an iQ5 optical system (Bio-Rad) and Ct values were exported as Excel files. Expression of *sod-1* was calculated using GAPDH as a reference and normalized to male control.

### SOD activity assay

SOD-1 enzyme activity on cytosolic fractions was assayed using a colorimetric kit by Cayman Chemicals (Ann Arbor, MI) according to the manufacturer’s specifications. Potassium cyanide, a SOD-1 inhibitor, was used to differentiate SOD-1 from SOD-2 activity.

### Statistical analysis

Results are expressed as the mean±standard error of the mean (SEM). Variation between sexes and treatments for O_2_^•−^ and SOD activity measurements was analyzed using two-way ANOVA with the Student–Newman–Keuls *post hoc* test for pairwise comparisons. Differences between non-normally distributed variables (qPCR and protein data) were analyzed by the Wilcoxon rank sum and an ANOVA on ranks with the Dunn’s *post hoc* test for pairwise comparisons. All statistical analyses were performed using either SigmaPlot v10.0 (Systat Software Inc. CA, USA), or SAS (SAS Institute Inc., NC, USA). Threshold for statistical significance was assumed at *P*≤0.05.

## Results

### ^•^NO decreases generation of O_2_^•−^ in male, but not female VSMC.

**Fig. 1:**
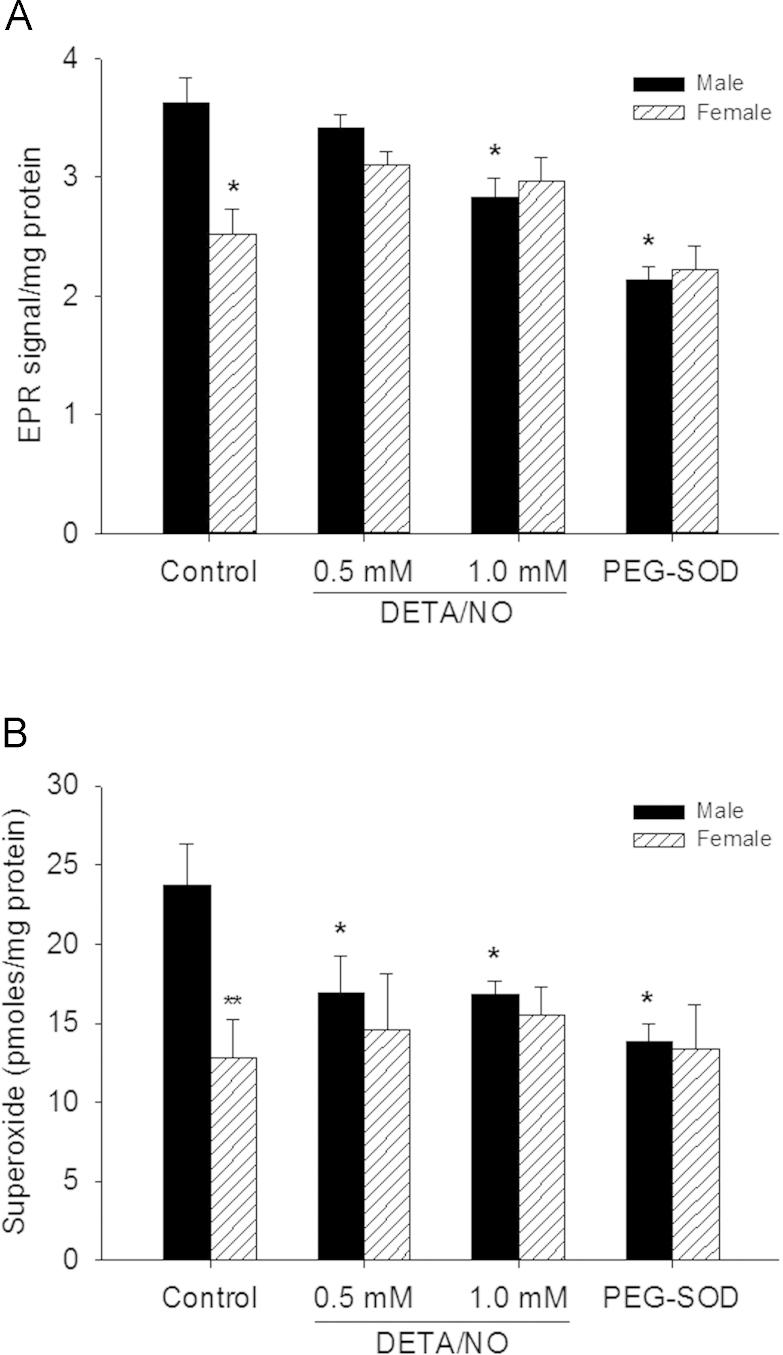
Superoxide (O_2_^•−^) levels are higher in male than female vascular smooth muscle cells (VSMC) at baseline and nitric oxide (^•^NO) decreases O_2_^•−^ levels in male, but not female, VSMC. (A) VSMC were treated with DETA/NO for 24 h, washed, incubated with 1-hydroxy-3-methoxy-carbonyl-2,2,5,5-tetramethylpyrrolidine (CMH) for 30 min, scraped and collected, then assessed via electron paramagnetic resonance (EPR) for the radical ^•^CM signal. Control cells were pre-treated with 50 units of pegylated superoxide dismutase (PEG-SOD). Two-way ANOVA analysis shows a significant interaction between sex and ^•^NO (*P*=0.003). EPR signal is higher in male vs. female VSMC at baseline (*P<0.001 vs. male control) but decreases in male VSMC at 24 h with DETA/NO treatment (*P<0.001 vs. male control, *N*=5). (B) VSMC were treated with DETA/NO for 24 h, washed, incubated with dihydroethidium for 1 h, scraped and collected, then 2-hydroxyethidium was quantified by HPLC. Control cells were pre-treated with PEG-SOD (50 units) for 24 h. Two-way ANOVA analysis shows a significant effect of ^•^NO (*P*=0.029). O_2_^•−^ levels measured by HPLC detection of 2-OH-E were higher in male vs. female VSMC (**P=0.002 vs. male control) but decreased in male VSMC at 24 h with DETA/NO treatment (*P<0.05 vs. male control, *N*=5).

To determine the effect of sex on O_2_^•−^ levels, EPR signal and HPLC analysis were used to quantify O_2_^•−^ (Fig. 1A and 1B). At baseline, ^●^CM EPR signal was higher in male compared to female VSMC (*P*<0.001), suggesting higher O_2_^•−^ levels in male VSMC. Following exposure to DETA/NO (1 mM), the ^•^CM EPR signal in male VSMC decreased 22% compared to untreated VSMC (Fig. 1A, *P*<0.001), suggesting a decrease in O_2_^•−^ levels. However, female VSMC show no significant change with increasing DETA/NO treatment (Fig. 1A). This interaction between sex and ^•^NO concentration is statistically significant (*P* = 0.003). VSMC pre-treated with PEG-SOD (50 U) also show a decrease in EPR signal, which suggests that the ^•^CM radical signal is due to O_2_^•−^ (Fig. 1A). To further validate these O_2_^•−^ findings, the O_2_^•−^-specific oxidation product of dihydroethidium, 2-hydroxyethidium (2-OH-E) was analyzed via HPLC (Fig. 1B), and a similar pattern emerged. At baseline, the levels of 2-OH-E levels were higher in male compared to female VSMC (*P* = 0.002). O_2_^•−^ generation in male VSMC decreased by 29% with increasing DETA/NO treatment (Fig. 1B, *P*<0.05). Again, female VSMC show no significant change with increasing DETA/NO treatment (Fig. 1B). As expected, VSMC exposed to PEG-SOD (50 U) showed a decrease in O_2_^•−^ levels. These data suggest that O_2_^•−^ levels are higher in male compared to female VSMC at baseline, but that ^•^NO affects O_2_^•−^ levels more in male compared to female VSMC.

### ^•^NO affects SOD-1 expression levels in male, but not female VSMC in a concentration- and time-dependent manner

**Fig. 2:**
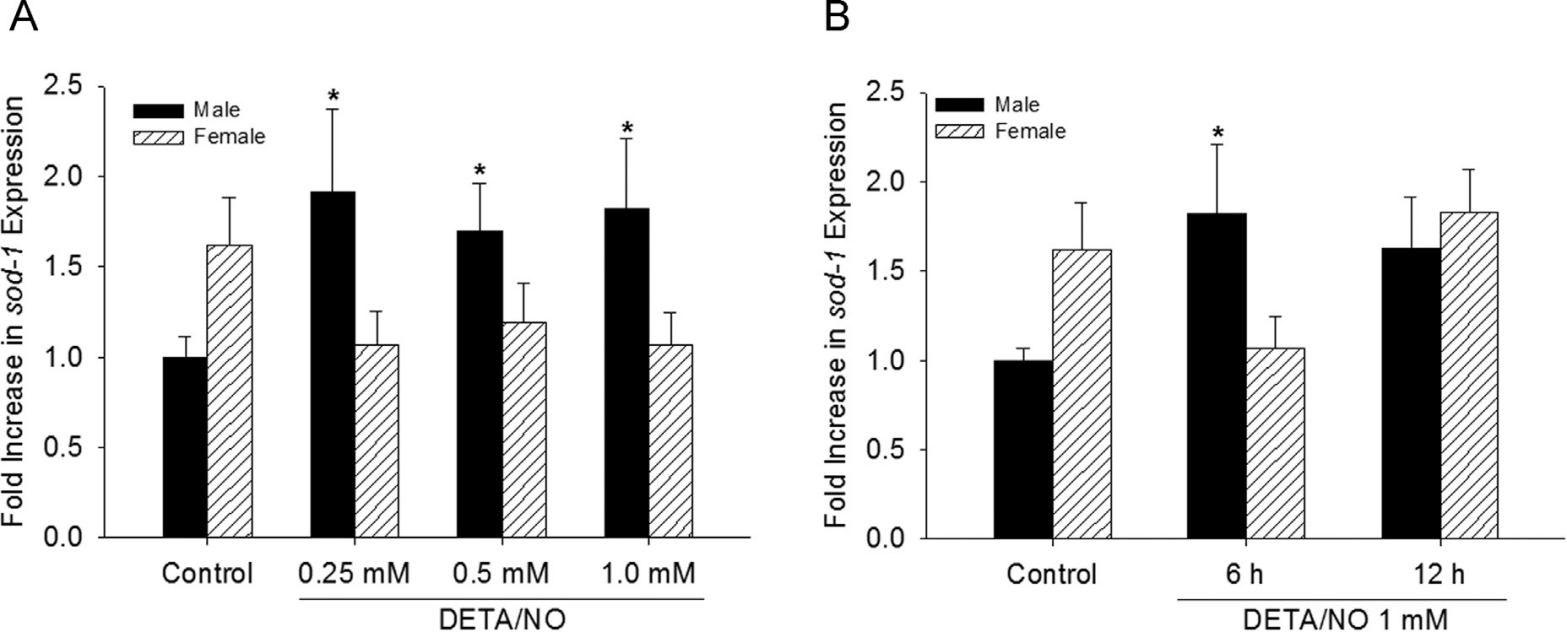
Nitric oxide (^•^NO) increases *sod-1* gene expression in male, but not female, VSMC. (A) Quantitative PCR showed *sod-1* gene expression is higher in females then males at baseline, and increases in male but not in female VSMC with DETA/NO treatment at 6 h (*P<0.05 vs. control, *N*=3 for males and *N*=4 for females). (B) Quantitative PCR showed *sod-1* gene expression time course. In male VSMC treated with DETA/NO *sod-1* increases at 6 h and starts to return to baseline by 12 h (*P<0.05 vs. control, *N*=8 for controls, *N*=3 for 6 and 12 h). In female VSMC, DETA/NO treatment has no significant effect at 6 and 12 h (*N*=7 for control, *N*=4 for 6 h, and *N*=3 for 12 h).

Given the differential O_2_^•−^ levels in male and female VSMC at baseline and following ^•^NO exposure, *sod-1* expression levels were examined via qPCR (Fig. 2). The data show a trend for females to have higher *sod-1* expression at baseline (1.9 fold). However, this trend did not reach statistical significance (Fig. 2; *P* = 0.07). On the other hand, expression of *sod-1* increased with all concentrations of DETA/NO tested at 6 h in male VSMC (Fig. 2A, *P*<0.05). Female VSMC showed no statistically significant change in *sod-1* expression with DETA/NO treatment (Fig. 2A). This interaction between sex and DETA/NO concentration is statistically significant (*P*=0.006). To explore whether the female response to ^•^NO could be delayed, *sod-1* expression was examined 12 h following exposure to DETA/NO (1 mM) (Fig. 2B). While *sod-1* expression increases to 2.1-fold at 6 h in male VSMC (*P*<0.05, Fig. 2B), it was not significantly different from control at 12 h (Fig. 2B). However, no significant increase in expression in females occurred with DETA/NO over 12 h (Fig. 2B). These data suggest *sod-1* gene expression in VSMC depend on sex and that ^•^NO has differential effects on regulating *sod-1* gene expression in male versus female VSMC.

### ^•^NO affects SOD-1 protein levels in male, but not female VSMC

**Fig. 3:**
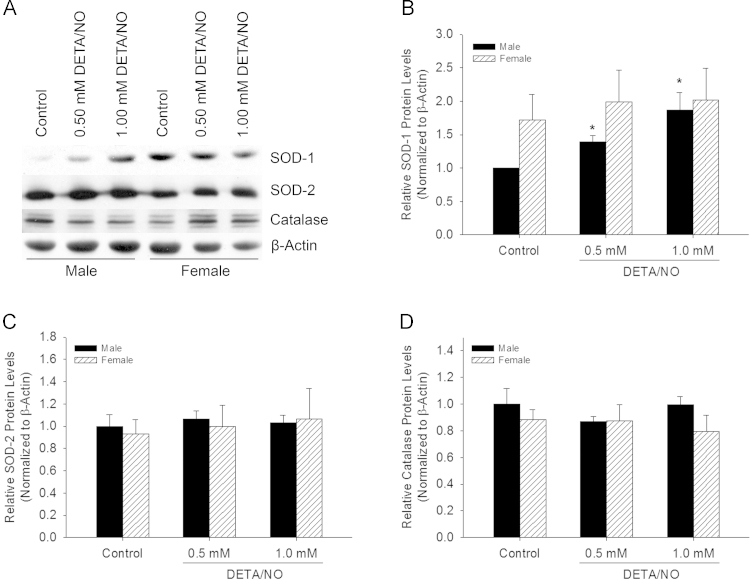
Nitric oxide (^•^NO) increases SOD-1 protein levels exclusively in male, but not female, VSMC. (A) Image of representative Western blots from male and female VSMC probed for SOD-1, SOD-2, catalase, and β-actin control. (B) Male and female densitometry data from Western blots probed for SOD-1. ^•^NO increases SOD-1 levels 1.9-fold in male VSMC at 24 h (*P<0.05 vs. male control; *N*=4). (C) Densitometry data from Western blots probed for SOD-2. SOD-2 protein levels do not differ at baseline or following DETA/NO treatment in male or female VSMC (*N*=4). (D) Densitometry data from Western blots probed for catalase. Catalase proteins levels do not differ at baseline for following DETA/NO treatment in male or female VSMC (*N*=4).

Given the differential effect on *sod-1* transcription between the sexes at baseline and after DETA/NO treatment, SOD-1 protein levels were assessed via Western blot analysis (Fig. 3A). Densitometry data of Western blots revealed that there was a trend toward higher SOD-1 protein levels at baseline in female versus male VSMC, but this difference did not reach statistical significance (*P*=0.4). When analyzed by sex, DETA/NO increased SOD-1 levels 1.9-fold in male VSMC compared to control (*P*<0.05, Fig. 3B). SOD-1 protein levels did not change in female VSMC following exposure to DETA/NO (Fig. 3B). In order to determine the relative contribution of SOD-1 in influencing these changes in oxidative stress, SOD-2 and catalase protein levels were also examined. SOD-2 (Fig. 3C) and catalase (Fig. 3D) protein levels were not different at baseline between the sexes, nor did they change significantly with increasing DETA/NO treatment in either sex. These data suggest that the action of ^•^NO in regulating O_2_^•−^ levels in VSMC is attributed mainly to changes in SOD-1 protein levels in male VSMC.

### ^●^NO affects SOD activity in male, but not female VSMC

**Fig. 4:**
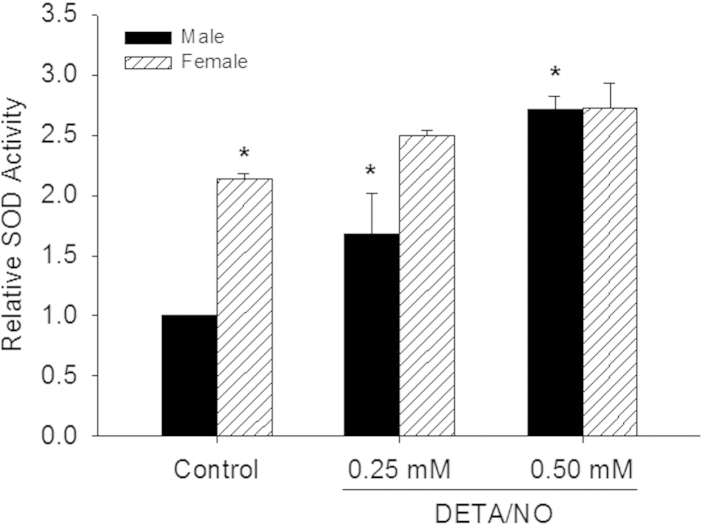
Superoxide dismutase (SOD) activity is higher at baseline in female vs. male vascular smooth muscle cells (VSMC), but nitric oxide (^•^NO) increases SOD activity in male, but not female, VSMC. VSMC were treated with DETA/NO for 24 h, washed, scraped and collected, then analyzed for SOD activity via colorimetric assay. Two-way ANOVA analysis shows a significant interaction between sex and ^•^NO (*P*=0.019). SOD activity was 2.1-fold higher in female VSMC at baseline compared to male VSMC (*P<0.001 vs. male control). DETA/NO increased SOD activity 2.7-fold in male VSMC (*P<0.001 vs. male control, *N*=6). SOD does not change in female VSMC with DETA/NO treatment (*N*=6).

Given the differences in O_2_^•−^ levels, *sod-1* gene expression, and SOD-1 protein levels following ^•^NO treatment between the sexes, we assessed SOD activity in both sexes at baseline and after ^•^NO exposure and normalized the data to male control for comparisons between the sexes (Fig. 4). Congruent with our previous findings in O_2_^•−^ levels and *sod-1* gene expression, female VSMC had 2.1 − fold higher SOD − 1 activity at baseline compared to male VSMC (*P*<0.001). Following treatment with DETA/NO, SOD activity increased 2.7-fold in male VSMC (*P*<0.001 vs. male control, Fig. 4). There was no DETA/NO -induced change in SOD-1 activity in female VSMC. This interaction between sex and ^•^NO concentration was statistically significant (*P* = 0.019). These data suggest that SOD activity in VSMC is based on sex and that ^•^NO modulates SOD activity more in male compared to female VSMC.

### ^•^NO differentially increases SOD-1 protein levels in male and female carotid arteries after injury.

**Fig. 5:**
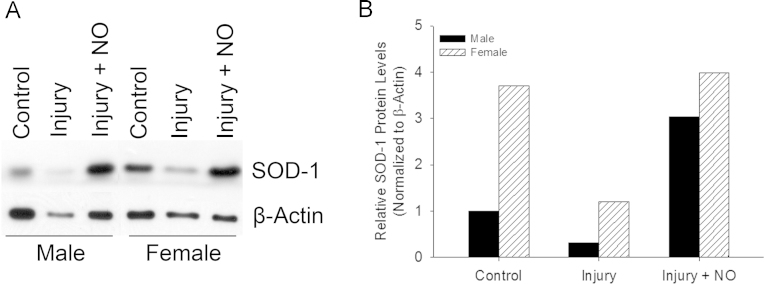
SOD-1 levels are higher in female compared to male carotid arteries but nitric oxide (^•^NO) increases superoxide dismutase-1 (SOD-1) protein levels above basal levels in male but not female carotid arteries*.* (A) Images of Western blots from male and female carotid arteries for SOD-1 and β-actin in control arteries, arteries harvested 3 days after balloon injury, and arteries harvested 3 days after balloon injury and treatment with ^•^NO (*N*=5 carotid arteries/treatment group). (B) Densitometry of SOD-1 protein levels from the Western blots of carotid artery lysates normalized to β-actin levels. SOD-1 protein levels were 3.7-fold higher in female carotid arteries compared to males. SOD-1 protein levels decreased following arterial injury in both sexes. ^•^NO increased SOD-1 protein levels 3.0-fold above control levels in males, but returned SOD-1 protein levels to control levels in females.

To determine if SOD-1 levels are effected by ^•^NO *in vivo*, SOD-1 protein levels were assessed via Western blot analysis from lysates isolated from carotid arteries that were balloon injured or injured and exposed to ^•^NO treatment (Fig. 5). At baseline, female carotid arteries exhibited higher SOD-1 protein levels compared to male carotid arteries (Fig. 5A). SOD-1 protein levels decreased 3 days after balloon injury in both sexes as compared to control arteries. Treatment with ^•^NO increased SOD-1 levels following arterial injury in both sexes compared to injury alone (Fig. 5A). Densitometry data of Western blots were normalized to β-actin and male control to compare data between the sexes (Fig. 5B). Female carotid arteries had 3.7-fold higher SOD-1 protein levels at baseline compared to male carotid arteries. Following arterial injury, SOD-1 protein levels decreased similarly in both sexes (69% vs. 68%, respectively). Following arterial injury and ^•^NO exposure, SOD-1 protein levels increased 3.0-fold higher then control levels in males, but returned to baseline levels in females (Fig. 5B). These data provide further evidence that SOD-1 levels differ at baseline between the sexes, and that ^•^NO exerts a differential effect on male and female SOD-1 regulation after injury *in vivo*.

## Discussion

Our present study shows that significant differences exist in O_2_^•−^ levels and SOD activity between male and female VSMC and carotid arteries at baseline. We also show that ^•^NO exposure affects the redox environment differently between the sexes in VSMC and carotid arteries. At baseline, O_2_^•−^ levels were higher in male VSMC and this corresponded to lower SOD activity, as compared to female VSMC. Basal SOD-1 protein levels and *sod-1* gene expression, both showed a trend to be higher in female than males. Following exposure to ^•^NO, O_2_^•−^ levels decreased in male VSMC and were associated with corresponding increases in *sod-1* gene expression, SOD-1 protein levels, and SOD activity. ^•^NO caused no such changes in female VSMC. A similar pattern was also observed in carotid arteries following arterial injury and ^•^NO exposure, with greater SOD-1 levels at baseline in female compared to male carotid arteries, a decrease in SOD-1 levels in both sexes after injury, but a much greater increase in SOD-1 levels in male versus female carotid arteries following ^•^NO exposure. Overall, our results provide evidence that regulation of the redox environment at baseline and following exposure to ^•^NO is sex-dependent in the vasculature.

This sex-based differential regulation of SOD-1 has implications in pro-proliferative states such as neointimal hyperplasia. SOD has been shown to decrease VSMC proliferation and migration *in vitro* [36], [37]. It has also been shown that overexpression of SOD-1 limits the development of neointimal hyperplasia *in vivo*. Kuo et al. showed that arteries in which SOD-1 was overexpressed developed 50% less neointimal hyperplasia compared to controls [29]. Muscoli et al. delivered a SOD mimetic to rats to inhibit balloon-induced neointimal hyperplasia [30]. On the other hand Hogg et al. showed that male VSMC proliferated and migrated more than female VSMC, and that ^•^NO inhibited proliferation and migration more effectively in male VSMC compared to female cells [21]. Thus, given that our laboratory that ^•^NO specifically increases SOD-1 levels both *in vitro* and *in vivo* in males, our current findings on the sex-based regulation of SOD-1 following exposure to ^•^NO may account for the sex-based difference in the inhibition of neointimal hyperplasia by ^•^NO [21], [31].

Interestingly, we observed differences in basal levels of O_2_^•−^ and SOD-1 activity between the sexes, with higher SOD activity in females and corresponding lower O_2_^•−^ levels. These data are consistent with the research showing that premenopausal women develop less CAD and have lower cardiovascular mortality [38]. In addition, several investigations are supportive of our overall finding. For example, Lam et al. showed that estrogen can directly impact ROS generation by increasing the availability of tetrahydrobiopterin, preventing eNOS uncoupling and ROS generation [39]. Stirone et al. showed that estrogen can increase eNOS gene expression while McNeill et al. demonstrated increased eNOS protein levels in endothelial cells and VSMC with 17β estradiol treatment [40], [41]. Arias-Loza et al. demonstrated that estrogen can directly regulate the subunits of NADPH oxidase, an enzyme responsible for ROS production, resulting in additional control of oxidative stress [42]. Most importantly, Strehlow et al. showed that 17β-estradiol specifically upregulates SOD expression and activity, resulting in the inhibition of ROS levels in VSMC [43]. Thus, our study demonstrating a sex-based differential in basal oxidative stress is supported by existing literature on the effects of estrogen. However, our study is distinct because we specifically link sex to differential regulation of the redox environment at baseline, following arterial injury, and following treatment with ^•^NO, and the severity of neointimal hyperplasia observed *in vivo*.

Bahnson et al. demonstrated the importance of ^•^NO-dependent regulation of SOD-1 in inhibiting VSMC proliferation through modulation of O_2_^•−^ levels [31]. DHE fluorescence serves as a general indicator for O_2_^•−^ levels and has been previously used to qualify and quantitate the extent of reactive oxidative species. Similar to our findings, Wedgwood et al. reported a concurrent 80% reduction in DHE fluorescence in VSMC exposed to ^•^NO [44]. However, Wedgwood et al. analyzed total DHE fluorescence which is not a specific measurement of O_2_^•−^. Though we report smaller decreases in O_2_^•−^ levels, our results are based on HPLC detection of 2-hydroxyethidium, the only validated, specific method for O_2_^•−^ measurement [45]. In addition, Wedgwood et al. assessed DHE fluorescence with the ^•^NO donor still present in the cells [44]. As ^•^NO readily reacts with O_2_^•−^, the reduction in DHE fluorescence could be confounded by O_2_^•−^ scavenging by ^•^NO, rather than be a consequence of redox protein regulation upon treatment. To account for this in our experimental design, the ^•^NO donor was completely removed and the cells thoroughly washed before exposing the cells to DHE. In doing so, the decrease in O_2_^•−^ in male VSMC is attributed to an increase in SOD-1 levels and activity, as opposed to a direct reaction of ^•^NO with O_2_^•−^. Nevertheless, a direct reaction between ^•^NO and O_2_^•−^ while the ^•^NO donor is present cannot be discounted. Moreover, Bahnson et al., have reported increased nitration in male VSMC treated with DETA/NO[31].

Our study is not without limitations. Firstly, in our O_2_^•−^ measurement experiments, we used PEG-SOD as a control. We showed that, as expected PEG-SOD decreased O_2_^•−^ levels in males. However, PEG-SOD failed to further decrease the O_2_^•−^ signal in females. Moreover, basal O_2_^•−^ levels in females were as low as the levels in males treated with exogenous PEG-SOD. A possibility for this phenomenon is that baseline levels of the O_2_^•−^ are already so low that the PEG-SOD added is not enough to cause a further reduction. Secondly, we analyzed the redox environment and the O_2_^•−^−SOD relationship using a variety of techniques. Whereas SOD-1 activity, O_2_^•−^ levels, and *sod-1* gene expression showed a statistically significant interaction between sex and ^•^NO, SOD-1 protein levels did not. This is probably due to the sample size used in the Western blot analysis and its intrinsic semi-quantitative nature, which may not have provided sufficient statistical power. When analyzed separately, we found that SOD-1 protein levels are higher upon DETA/NO treatment, consistent with previous findings by Bahnson et al. [31] This effect was not observed in females. In this work, we show significant changes in gene expression, activity, and O_2_^•−^ levels in male VSMC but not in female VSMC with a significant interaction between sex and ^●^NO variables. In addition, we focused primarily on an analysis of the impact of ^●^NO on SOD-1; therefore, the interplay between additional protein factors as well as the dynamics between other cell types (e.g., endothelial cells, adventitial fibroblasts) was excluded from this study. However, we have previously shown that ^•^NO treatment has no effect on SOD-1 in adventitial fibroblasts and endothelial cells from male rats [31]. While SOD-2 and catalase were examined to rule out the influence of other proteins *in vitro*, we did not investigate the role of other redox enzymes, such as NADPH oxidases or glutathione peroxidases. Furthermore, analysis outside of a pathophysiologic model *in vitro* potentially obfuscates the true roles of additional antioxidant proteins in concert. While a comprehensive *in vivo* study is outside the scope of this project, we still show evidence that the expression of SOD-1 is differentially affected at baseline and by ^•^NO based on sex both *in vitro* and *in vivo*. Although our study does not explore a direct correlation of SOD-1 levels and neointimal hyperplasia *in vivo* based on sex, we have previously shown that ^•^NO is less effective at inhibiting neointimal hyperplasia in male SOD-1 knockout mice [31].

In conclusion, we show that regulation of the redox environment in the vasculature at baseline and following ^•^NO treatment is sex dependent. These data are consistent with our laboratory’s previous finding of differential efficacy on ^•^NO on the inhibition of neointimal hyperplasia based on sex [21]. By furthering our understanding of how sex impacts the vasculature, better therapies can be developed for both sexes, bring personalized medicine close to reality.

## Conflicts of interest

This work was supported by the Women’s Health Research Institute and the Society for Vascular Surgery Foundation. Funding sources had no influence in the conduct of the research or preparation of the article. The authors report no proprietary or commercial interest in any product mentioned or concept discussed in this article.

## Appendix A Supplementary material

Supplemental Fig. 1 Verification of the sex of male and female vascular smooth muscle cells (VSMC) using polymerase chain reaction (PCR). Presence of secondary bands in male samples (arrow) denotes positive verification of Y-chromosome. Lane 1: 100 bp ladder, Lane 2: Male (100 ng template DNA), Lane 3: Male (200 ng template DNA), Lane 4: female (100 ng template DNA), Lane 5: female (200 ng template DNA), Lane 6: negative control (*n*=1).
