# Supplementary figures and images for: Sex-based differential regulation of oxidative stress in the vasculature by nitric oxide
Source: Redox Biol. 2015 Jan 13;4:226–33. doi: 10.1016/j.redox.2015.01.007 (PMC4803798; doi:10.1016/j.redox.2015.01.007)

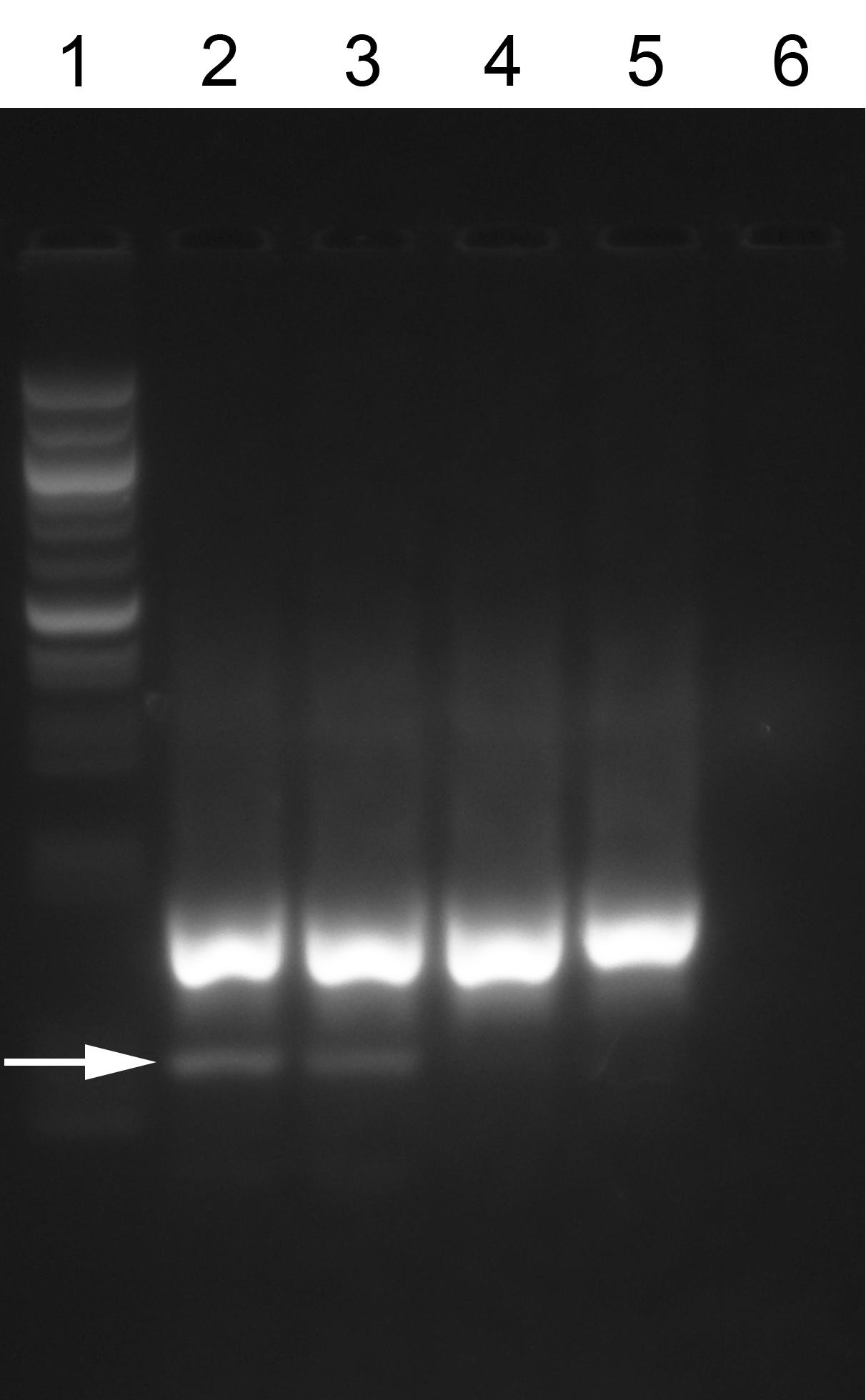

Supplement: Supplemental Fig. 1 — Verification of the sex of male and female vascular smooth muscle cells (VSMC) using polymerase chain reaction (PCR). Presence of secondary bands in male samples (arrow) denotes positive verification of Y-chromosome. Lane 1: 100 bp ladder, Lane 2: Male (100 ng template DNA), Lane 3: Male (200 ng template DNA), Lane 4: female (100 ng template DNA), Lane 5: female (200 ng template DNA), Lane 6: negative control (n=1). [file mmc1.zip › FigS1.tif]
